# PRR enhances anti-tumor immunity and suppresses colitis by promoting the development and survival of naive T and iNKT cells

**DOI:** 10.3389/fimmu.2025.1566935

**Published:** 2025-12-10

**Authors:** Akihiro Shimba, Satoru Munakata, Shinya Abe, Guangwei Cui, Ryoma Kato, Masaki Miyazaki, Atsuhiro Ichihara, Koichi Ikuta

**Affiliations:** 1 Laboratory of Immune Regulation, Department of Virus Research, Institute for Life and Medical Sciences, Kyoto University, Kyoto, Japan; 2 Division of Integrated High-Order Regulatory Systems, Center for Cancer Immunotherapy and Immunobiology, Graduate School of Medicine, Kyoto University, Kyoto, Japan; 3 Department of Homeostatic Medicine, Medical Research Laboratory, Institute for Integrated Research, Institute of Science Tokyo, Tokyo, Japan; 4 Jiangsu Key Laboratory of Tissue Engineering and Neuroregeneration, Key Laboratory of Neuroregeneration of Ministry of Education, Co-innovation Center of Neuroregeneration, Nantong University, Nantong, China; 5 Laboratory of Immunology, Institute for Life and Medical Sciences, Kyoto University, Kyoto, Japan; 6 Department of Endocrinology and Hypertension, Tokyo Women’s Medical University, Tokyo, Japan

**Keywords:** (pro)renin receptor, lysosome, mitochondria, iNKT, colitis, anti-tumor immunity

## Abstract

The (pro)renin receptor (PRR) is a multifunctional transmembrane protein that enhances β-catenin/TCF1 signaling and V-ATPase-mediated lysosomal acidification. Emerging evidence indicates that it may also regulate potential roles in regulating T cell development, survival, and immune responses. Here, we demonstrated that PRR promotes the maturation and survival of T cells within the thymus. In particular, PRR-deficient mice exhibited a significant reduction in iNKT cells in the thymus and periphery. PRR promoted the energy synthesis process in mitochondria, as evidenced by increased mitochondrial amount and membrane potential. This phenomenon was accompanied by an increase in TCF1 expression and lysosomal acidification. Furthermore, PRR enhanced the survival of naive T and iNKT cells in the periphery, while simultaneously suppressing inflammatory cytokine-producing T cells, thereby preventing colitis. In contrast, PRR enhanced resistance against tumor growth by increasing the number of tumor-infiltrating Th1 and iNKT cells, which in turn promoted NK cell recruitment. This study indicates that PRR is critical for supporting T cell maintenance, suppressing excessive inflammation, and enhancing anti-tumor immunity.

## 1 Introduction

The (pro) renin receptor (PRR), encoded by *Atp6ap2*, is a multifunctional single transmembrane protein that is ubiquitously expressed (1). PRR was initially identified as a receptor for (pro) renin, which degrades angiotensinogen. In addition, PRR serves as an accessory protein in complexes with the vacuolar-type ATPase (V-ATPase) and Wnt receptors. V-ATPase is a proton pump that regulates the cellular pH and organelle acidification. This is essential for intracellular transport by the endoplasmic reticulum and for organelle clearance by lysosomal degradation (2). V-ATPase dysfunction results in the accumulation of protein aggregates and damaged organelles, which ultimately leads to cell death (3). Wnt/β-catenin/TCF1 signaling regulates the cell cycle and mitochondrial biogenesis (4). Moreover, TCF1 plays a significant role in T cell differentiation within the thymus, T cell survival, and helper T cell function (5–7). PRR full-knockout mice exhibit embryonic lethality (8). In addition, previous studies have indicated that PRR is critical for the survival of cardiomyocytes, podocytes, and double-negative (DN) thymocytes, as evidenced by cell-specific deletion of PRR in mice (9–11). These findings indicate that PRR supports the survival and function of T cells, potentially by regulating organelle quality.

PRR may regulate mitochondrial quality control through Wnt/TCF1-mediated mitochondrial biogenesis and lysosome-dependent mitochondrial clearance pathways. Mitochondria efficiently produce ATP via acetyl-CoA oxidation during the TCA cycle and oxidative phosphorylation (OXPHOS) (12). The metabolic state of T cells varies across different stages of T cell activation and among different subsets (13). Naive T cells exhibit metabolic quiescence and depend on OXPHOS for prolonged survival (14). Conversely, activated T cells elevate glycolytic flux for differentiation and cell proliferation, because aerobic glycolysis induces rapid ATP production (13, 14). Therefore, it is important to control the metabolic balance between OXPHOS and glycolysis to maintain T cells and ensure optimal immune responses. Dysfunction of mitochondrial biogenesis and autophagy results in a reduction in peripheral T cells and an enhancement in the development of inflammatory cytokine-producing T cells (15–18). Consequently, the malfunction of mitochondria and lysosomes increases the susceptibility to tumors while exacerbating inflammatory diseases such as colitis and systemic lupus erythematosus (15, 17, 19–22). This indicates that PRR regulates mitochondrial quality through V-ATPase and TCF1, thereby promoting the maintenance of peripheral T cells and inhibiting the development of inflammatory diseases.

Mitochondrial respiration and autophagy play pivotal roles in the development of invariant natural killer T (iNKT) cells. iNKT cells are a group of innate-like T lymphocytes that express invariant TCRs that recognize lipid antigens presented on the MHC class I-like molecule CD1d (23). Similar to conventional T cells, iNKT cells develop in the thymus from CD4^+^CD8^+^ double-positive (DP) thymocytes through positive selection (23). TCR stimulation and inflammatory cytokines rapidly induce the production of several cytokines, such as IFN-γ and IL-4, from iNKT cells, thereby enhancing protection against tumor growth and viral infection, while mitigating some inflammatory diseases, such as colitis and autoimmune arthritis (24–28). It has been demonstrated that the inhibition of mitochondrial OXPHOS by oligomycin results in impaired survival and cytokine production in iNKT cells (29). Furthermore, T cell-specific ablation of Rieske iron-sulfur protein (RISP), an essential subunit of mitochondrial complex III, drastically reduces iNKT cells (30). Moreover, deficiency of autophagy-related gene 7 (Atg7) significantly impairs iNKT cell development and reduces Bcl-2 expression (31). These findings indicate a regulatory role for PRR in the development of iNKT cells, potentially through the control of mitochondrial and lysosomal activity. Taken together, PRR may promote the survival and function of various T cell subsets, thereby enhancing protection against infection and tumors while suppressing inflammatory diseases.

To investigate the role of PRR in the development and function of conventional T and iNKT cells, we analyzed T cell-specific PRR-deficient mice. Our findings revealed a significant decline in the numbers of conventional T cells and iNKT cells in PRR-deficient mice. PRR maintained Bcl-2 expression and mitochondrial membrane potential, thereby supporting the survival of naive T cells. Conversely, PRR inhibited the generation of CD44^high^ effector T cells, thereby inhibiting colitis. Moreover, PRR facilitated the recruitment of IFN-γ-producing T and iNKT cells, resulting in the inhibition of tumor growth. In conclusion, this study demonstrated that PRR plays a crucial role in maintaining immune homeostasis by promoting the survival of non-pathogenic T cells and inhibiting the overproduction of pathogenic and inflammatory T cells. These findings suggest that PRR may serve as a potential therapeutic target for the prevention of tumors and autoimmune diseases.

## 2 Materials and methods

### 2.1 Mice

C57BL/6 (CD45.2) mice were obtained from Japan SLC. B6.CD45.1 congenic, CD4-Cre (32), Rosa26-YFP (33), and *Rag2*^−/−^ mice were used in this study. *Atp6ap2*^flox^ mice were provided by Dr. Atsuhiro Ichihara of Tokyo Women’s Medical University (9). CD4-CreERT2 mice were provided by Dr. Masaki Miyazaki of Kyoto University (34). The mice were maintained under specific pathogen-free conditions at the Experimental Research Center for Infectious Diseases at the Institute for Life and Medical Sciences, Kyoto University, and were used according to a protocol approved by the Animal Experimentation Committee of the Institute. Mice were maintained in groups under controlled humidity, temperature, and light (12-hour light/12-hour dark cycles). Food and water were provided *ad libitum*. All procedures were performed under sevoflurane anesthesia to minimize the suffering of the animals. Experiments were performed on male and female mice at 6–14 weeks of age. Each pair of control and mutant mice of the same sex from the same litter was analyzed simultaneously, and the data were pooled from multiple mixed-sex pairs from different litters.

### 2.2 Cell preparation

Bone marrow (BM) cells were obtained from the tibia and femur of the mice. Cells from the thymus, spleen, and lymph nodes (LNs) were mechanically prepared by crushing the organs using a cell strainer. To prepare liver cells, the liver was crushed using a 40-μm cell strainer and separated by centrifugation through 40% Percoll (GE Healthcare). To prepare lamina propria (LP) cells, the small intestine and colon, from which Peyer’s patches were removed, were opened longitudinally and incubated in PBS containing 5 mM EDTA (Nacalai Tesque) at 37 °C for 30 min. After incubation, the tissues were washed with PBS to remove the epithelial cells and debris. The remaining tissues were minced with scissors and incubated for 1 hour at 37 °C in RPMI 1640 (Nacalai Tesque) medium containing 10% fetal bovine serum (FBS: ICN Biomedicals), 1.25 mg/ml collagenase D (Roche), and 50 μg/ml DNase I (Worthington). The digested tissues were filtered through a 40-μm strainer. Leukocytes were separated by centrifugation using a 40% Percoll solution. To prepare lung lymphocytes, the lungs were minced and incubated for 1 h at 37 °C in RPMI 1640 medium containing collagenase D and DNase I. The digested tissues were passed through a 40-μm strainer and separated by centrifugation through 40% Percoll.

### 2.3 Flow cytometry and antibodies

Cells were stained with antibodies for 20 min at 4 °C in PBS containing 0.05% NaN_3_ (Nacalai Tesque) and 0.2% bovine serum albumin (BSA: Nacalai Tesque). The following fluorescent dye- or biotin-conjugated antibodies against specific proteins for mice were used: CD3ε (145-2C11), TCRβ (H57-597), γδTCR (GL3), CD69 (H1.2F3), CCR7 (4B12), CD62L (MEL-14), NK1.1 (PK136), CD49a (HMα1), CD49b (DX5), KLRG1 (2F1), Nkp46 (29A1.4), PD-1 (29F.1A12), CD45 (30-F11), CD45.1 (A20), CD45.2 (104), Flt3 (A2F10), Sca-1 (E13-161.7), CD117 (2B8), α4β7 (DATK37), CD19 (6D5), CD11c (N418), CD11b (M1/70), Gr1 (RB6-8C5), Ly6G (1A8), IL-17A (TC11-18H10.1), phosphorylated LCK Tyr394 (A18002D), Ki-67 (SoIA15), PLZF (9E12), and IgG1 isotype (MOPC-21) from BioLegend; CD4 (RM4.5), CD8α (53-6.7), Foxp3 (FJK-16s), and CD25 (PC61) from Thermo Fisher Scientific; CD44 (IM7) and IFN-γ (XMG1.2) from TONBO Biosciences; Bcl-2 (A19-3) and TCF1 (S33-966) from BD pharmingen; CD1d-tetramer from NIH Tetramer Core Facility. Viable cells were analyzed using an LSRFortessa or FACSCanto II flow cytometer (BD Biosciences) and FlowJo software. The values in the quadrants, gated areas, and interval gates indicate the percentage of cells in each population. To analyze the TCR Vβ repertoire, thymocytes and splenocytes were stained with monoclonal antibodies specific for the different Vβ chains (Mouse Vβ TCR Screening Panel, BD Biosciences). For intracellular staining of Bcl-2, Foxp3, and TCF1, lymphocytes were first stained for surface antigens, fixed, and permeabilized. Finally, the cells were stained with the appropriate antibodies using the Foxp3 Staining Buffer Set (eBioscience). For intracellular staining of cytokine production, cultured CD4 T cells were fixed, permeabilized, and stained with the appropriate antibodies using IC Fixation Buffer (eBioscience). For intracellular staining of phosphorylated LCK, thymocytes were fixed, permeabilized, and stained with the appropriate antibodies using IC Fixation Buffer. For mitochondrial or lysosomal staining, cells were incubated with PBS containing 500 nM Mito Tracker Green (Invitrogen), 5 µM Mito Sox Red (Invitrogen), 2 µmol/l JC-1 Dye (Dojindo), or 500 nM LysoSensor Yellow/Blue (Invitrogen) for 30 min at 37 °C.

### 2.4 Cell isolation

For Th subset induction, naive CD4 T cells were purified from lymph nodes using a MagniSort mouse CD4 naive T cell enrichment kit (Thermo Fisher Scientific). CD45RB^high^CD4^+^TCRβ^+^ cells from the LNs were sorted and transferred into *Rag2*-deficient mice, whereas CD1d-tetramer^+^TCRβ^+^ cells from the spleen and LNs were sorted and transferred into PRR-deficient mice using a FACSAria II cell sorter (BD Biosciences).

### 2.5 Cell culture

Cells were cultured in RPMI 1640 medium containing 10% FBS, 50 μM 2-mercaptoethanol (Kishida Chemical Co), 10 mM HEPES (pH 7.4, Nacalai Tesque), 100 U/ml penicillin (Nacalai Tesque), and 100 μg/ml streptomycin (Nacalai Tesque) at 37 °C. Naive CD4^+^ T cells were isolated from LNs and cultured with 6 μg/ml plate-bound anti-CD3 antibody (2C11, Bio X Cell) and 6 μg/ml plate-bound anti-CD28 antibody (37.51, Bio X Cell) in the presence of IL-2 (20 ng/ml, BioLegend), IL-12 (20 ng/ml, BioLegend), and anti-IL-4 antibody (5 ng/ml, BioLegend) for 5 days for Th1 differentiation. For Th17 differentiation, cells were cultured in the presence of IL-6 (20 ng/ml, BioLegend), IL-1β (20 ng/ml, BioLegend), IL-23 (30 ng/ml, BioLegend), TGF-β (10 ng/ml, BioLegend), anti-IFN-γ antibody (5 ng/ml, BioLegend), and anti-IL-4 antibody (5 ng/ml) for 4 days. For Treg differentiation, cells were cultured in the presence of TGF-β (10 ng/ml), IL-2 (20 ng/ml), anti-IL-4 antibody (5 ng/ml), and anti-IFN-γ antibody (5 ng/ml) for 5 days. The cells were fixed, permeabilized, and stained with antibodies against specific cytokines. To detect cytokine production, the cells were restimulated with PMA (50 ng/ml, Cayman) and ionomycin (2 μg/ml, Cayman) for 3 hours in the presence of brefeldin A (10 μg/ml). For proliferation analysis, isolated naive CD4 T cells were labeled with cell proliferation dye (CPD) eFluor 450 (5 μM, eBioscience) in PBS at 37 °C for 10 minutes and stimulated in the presence of 6 μg/ml plate-bound anti-CD3 antibody and 6 μg/ml plate-bound anti-CD28 antibody in the presence of IL-2.

### 2.6 Tamoxifen treatment

To prepare a solution of 20 mg/ml tamoxifen (Fujifilm Wako Pure Chemical Corporation), 20 mg of the drug was dissolved in 1 ml corn oil (Sigma-Aldrich). Mice were administered a 100 μl solution containing 2 mg of tamoxifen intraperitoneally at 24-hour intervals for 5 days. The mice were analyzed two weeks after the initial administration.

### 2.7 Mixed bone marrow chimera

To generate BM chimeric mice, 8-week-old CD45.1 congenic mice were irradiated at a dose of 9.0 Gy. Irradiated mice were reconstituted with a 1:1 mixture of 5 × 10^6^ BM cells from *Atp6ap2*^fl/fl^ (CD45.1^+^CD45.2^+^) and *CD4*^Cre^*Atp6ap2*^fl/fl^ (CD45.2^+^) mice via intravenous injection. The mice were analyzed 8 weeks after transplantation.

### 2.8 Metabolic profiling by modified SCENITH method with OP-puromycin

The cells were plated at a density of 2 × 10^6^ cells/ml in 96-well plates and cultured for 45 minutes at 37°C and 5% CO2 with control (Co), 2-deoxy-D-glucose (DG; 100 mM; Nacalai Tesque Inc.), oligomycin (O; 1 μM; Selleck Biotech), or a combination of both drugs (DGO). O-propargyl (OP)-puromycin (20 μM) from the Click-&-Go Plus 488 OPP Protein Synthesis Assay Kit (Click Chemistry Tools) was added to the culture medium for 30 min. The cells were then washed with cold PBS and incubated with primary conjugated antibodies against different surface markers for 20 min at 4°C in staining buffer (PBS containing 0.2% BSA). Following washing with staining buffer, the cells were fixed and permeabilized using the Foxp3 Staining Buffer Set (eBioscience). OP-puromycin incorporation was quantified using the Click-&-Go Plus 488 OPP Protein Synthesis Assay Kit and flow cytometry, as previously described (35).

### 2.9 Induction of colitis by adoptive transfer of T cells

CD45RB^high^CD4^+^ cells were sorted from the LNs of control or CD4-Cre PRRcKO mice, and 4 × 10^5^ cells were transferred intraperitoneally into *Rag2*-deficient mice. The mice were monitored weekly for weight loss and were subsequently analyzed on day 28.

### 2.10 DSS-induced colitis and adoptive transfer of iNKT cells

Sorted CD1d-tetramer^+^TCRβ^+^ cells were cultured with 6 μg/ml plate-bound anti-CD3 antibody and 6 μg/ml plate-bound anti-CD28 antibody in the presence of IL-2 (20 ng/ml). After 3 days, 5 × 10^5^ iNKT cells were transferred into the control and *PRR*-deficient mice. Mice were administered 4% (w/v) DSS (dextran sulfate sodium, MW 36,000~50,000, MP Biomedicals) in drinking water for 7 days from the day iNKT cells were transferred. The mice were then monitored for weight changes.

### 2.11 B16-F10 melanoma and adoptive transfer of iNKT cells

The B16-F10 melanoma cell line was cultured in high-glucose-containing Dulbecco’s modified Eagle’s medium (Nacalai Tesque) supplemented with 10% FBS, penicillin (100 U/ml), streptomycin (100 μg/ml), and 2 mM L-glutamine (Nacalai Tesque). A single-cell suspension of B16-F10 cells was prepared in PBS, and 1 × 10^6^ cells were injected subcutaneously into the recipient mice. On day 14, the tumor volume was measured, and lymphocytes were isolated and analyzed. Sorted CD1d-tetramer^+^TCRβ^+^ cells were cultured with 6 μg/ml plate-bound anti-CD3 antibody and 6 μg/ml plate-bound anti-CD28 antibody in the presence of IL-2 (20 ng/ml), IL-12 (20 ng/ml), and IL-18 (20 ng/ml: BioLegend) for 3 days. Following stimulation, 5 × 10^5^ iNKT cells were transferred into the control and *PRR*-deficient mice. Simultaneously, 1 × 10^6^ B16-F10 cells were injected subcutaneously into the recipient mice.

### 2.12 Histology

Freshly harvested thymuses were fixed in 4% paraformaldehyde (PFA, Fujifilm) at 4°C for 7 h, after which the solution was replaced with 10%, 20%, and 30% sucrose solutions (Fujifilm) in PBS at 4°C for 3 days. The tissues were then embedded in O.T.C. compound (Sakura Finetek Japan) and frozen in cooled hexane (Fujifilm). Sections (10 μm thick) were prepared using a Leica cryostat and stained with hematoxylin and eosin (Wako). The thymic cortex appeared darker and more basophilic due to the presence of densely packed immature thymocytes. In contrast, the medulla looked lighter and more eosinophilic due to the presence of fewer lymphocytes and more epithelial cells.

### 2.13 Statistical analysis

All data are presented as mean ± SD. Two samples were compared using an unpaired two-tailed Student’s *t*-test. For multiple group comparisons, one-way analysis of variance (ANOVA) with Tukey’s multiple comparison tests and two-way ANOVA with Bonferroni’s and Tukey’s multiple comparison tests were performed. The asterisks in all figures indicate the following: * *p* < 0.05, ** *p* < 0.01, and *** *p* < 0.001. Statistical analyses were performed using Prism 8 (GraphPad).

## 3 Results

### 3.1 PRR promotes TCR signaling and survival of thymocytes after positive selection

**Figure 1:**
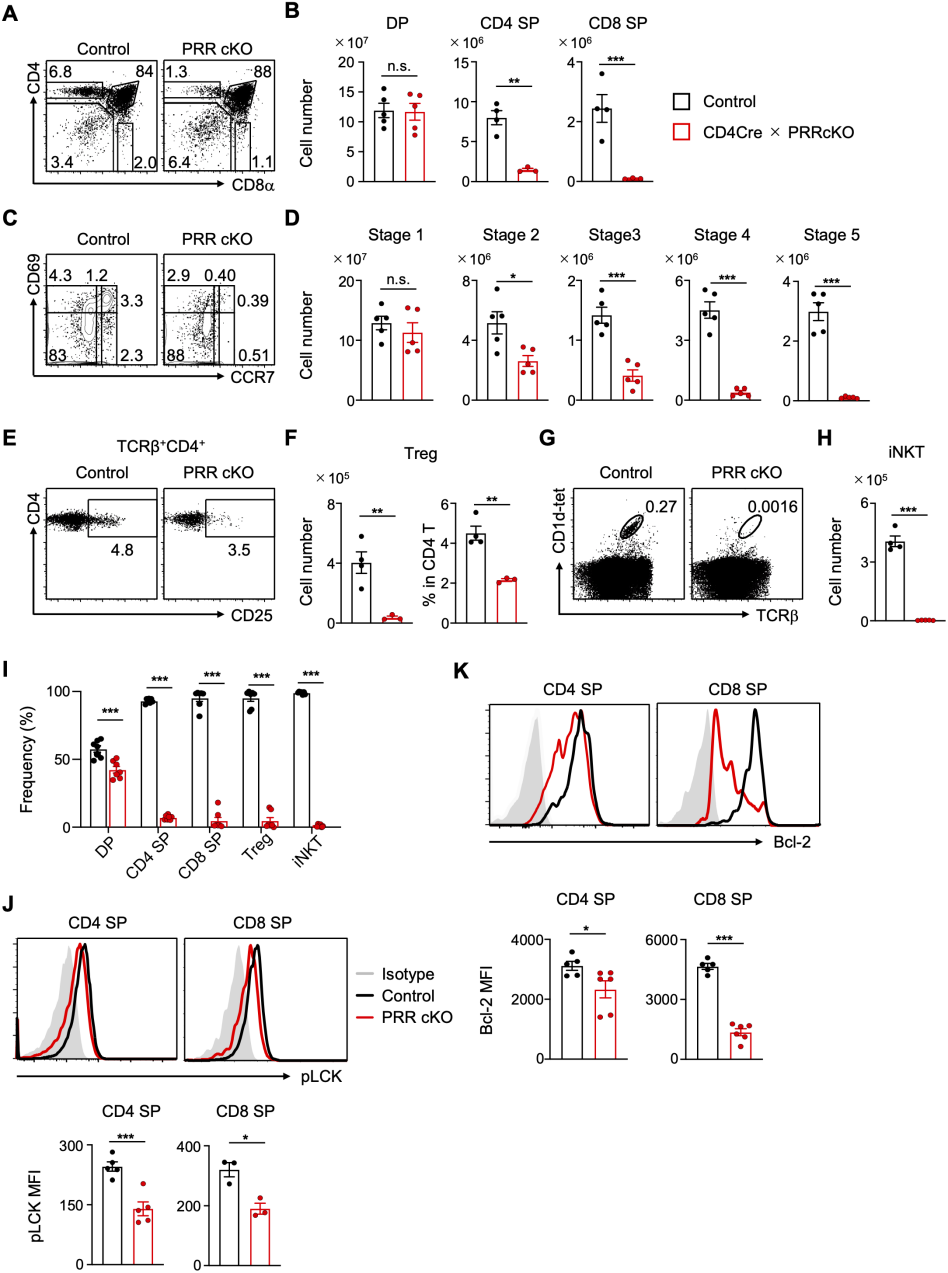
T cell development and survival after positive selection are impaired in PRR-deficient mice. **(A)** Representative FCM plots of CD4 and CD8 expression in the thymocytes of control (*CD4Cre(-) Atp6ap2*^+/Y^) and CD4-Cre PRRcKO (*CD4Cre(+) Atp6ap2*^flox/Y^) mice. **(B)** Numbers of DP (CD4^+^CD8^+^) (n = 5), CD4 SP (CD4^+^CD8^−^TCRβ^+^), and CD8 SP (CD8^+^CD4^−^TCRβ^+^) (n = 3-4) thymocytes in control and CD4-Cre PRRcKO mice. **(C)** Representative FCM plots of CD69 and CCR7 expression in thymocytes of control and CD4-Cre PRRcKO mice. Thymocytes mature through stage 1 (CCR7^−^CD69^−^), 2 (CCR7^−^CD69^+^), 3 (CCR7^low^CD69^+^), 4 (CCR7^high^CD69^+^), and 5 (CCR7^high^CD69^−^). **(D)** Number of thymocytes at maturation stages in **(C)** (n = 5). **(E)** Representative FCM plots of CD4 and CD25 expression in CD4^+^TCRβ^+^ thymocytes of control and CD4-Cre PRRcKO mice. **(F)** Number of CD25^+^CD4^+^TCRβ^+^ cells and frequency of Tregs relative to CD4 SP cells in the thymus of control and CD4-Cre PRRcKO mice (n = 3-4). **(G)** Representative FCM plots of CD1d-tetramer and TCRβ staining in thymocytes of control and CD4-Cre PRRcKO mice. **(H)** Number of iNKT (CD1d-tetramer^+^TCRβ^+^) cells in the thymus of control and CD4-Cre PRRcKO mice (n = 4-5). **(I)** Lethally irradiated WT mice (CD45.1) were transferred with a 1:1 mixture of control (*CD4Cre(-) Atp6ap2*^+/Y^, CD45.1/2) and CD4-Cre PRR cKO (*CD4Cre(+) Atp6ap2*^flox/Y^, CD45.2) bone marrow cells and analyzed 8 weeks post-transfer. Relative frequencies of DP, CD4 SP, CD8 SP, Treg, and iNKT cells in control and CD4-Cre PRR cKO mice (n = 7). **(J)** Representative FCM histogram and mean fluorescence intensity (MFI) of intracellular staining of phosphorylated LCK in CD4 SP (n = 5) and CD8 SP (n = 3) thymocytes of control and CD4-Cre PRRcKO mice. **(K)** Representative FCM histogram and MFI of Bcl-2 expression in CD4 SP and CD8 SP thymocytes of control and CD4-Cre PRRcKO mice (n = 5-6). Data are the mean ± SD with Student’s *t*-test and pooled from at least two independent experiments. ***p < 0.001; **p < 0.01; *p < 0.05; and n.s., not significant.

To address whether PRR controls thymocyte development after the DP stage, we analyzed CD4-Cre PRR cKO (CD4-Cre × *Atp6ap2*^flox/Y^ or *Atp6ap2*^flox/flox^) mice. The number of DP thymocytes remained unaltered in CD4-Cre PRR cKO mice, whereas the number of CD4 or CD8 single-positive (SP) thymocytes was significantly reduced (**Figures 1A, B**). This indicates that PRR supports thymocyte maturation and survival *in vivo*. To further examine the effects of PRR on T cell selection, thymocytes were fractionated according to their maturation stages by staining for CD69 and CCR7 (**Figures 1C, D**) (36). A reduction in thymocytes was observed in CD4-Cre PRR cKO mice at stage 2 (**Figure 1D**), indicating that PRR-deficient thymocytes could not develop because of defects in positive selection. Consistent with the reduction in mature T cells, the medullary region of the thymus was markedly smaller in CD4-Cre PRR cKO mice (**Supplementary Figure S1A**). To analyze the effect of a failure in positive selection on the TCRβ chain repertoire in CD4-Cre PRR cKO mice, we examined the frequency of each T cell population bearing different TCRβ chains. There was no clear trend in the TCRβ repertoire, suggesting that PRR enhances thymocyte survival uniformly (**Supplementary Figure S1B**).

Following the DP stage, regulatory T (Treg) and iNKT cells develop and diverge from conventional αβ T cells (23, 37). Treg cells were also significantly decreased in the thymus of CD4-Cre PRR cKO mice (**Figures 1E, F**). In addition, the frequency of Treg cells relative to the total CD4 SP cells decreased (**Figure 1F**), suggesting that PRR promotes the differentiation of Treg cells. Furthermore, iNKT cells were almost entirely absent from the thymus (**Figures 1G, H**). To investigate whether PRR is essential for the differentiation or early development of iNKT cells, we analyzed their developmental pathway. PLZF was not expressed in the iNKT cells of CD4-Cre PRR cKO mice (**Supplementary Figure S1C**), whereas the frequency of the most immature CD69^+^CD24^+^ iNKT cells was slightly higher than that in control mice (**Supplementary Figures S1D, E**). This suggests that PRR is important for the maturation of iNKT cells from the premature stage. In addition, the number of γδ T cells increased in CD4-Cre PRR cKO mice (**Supplementary Figure S1F**). As a drastic reduction in αβ T cells in the thymus increases γδ T cells through abundant cytokines (38), this suggests that the increase in γδ T cells in CD4-Cre PRR cKO mice was due to the reduction in αβ T cells. Taken together, these results suggest that PRR is critical for the development of mature T cell subsets, particularly iNKT cells.

To investigate whether PRR exerts an intrinsic effect on thymocyte maturation and iNKT cell development, we conducted experiments using mixed competitive bone marrow chimeras (**Figure 1I**). In host mice, the number of thymocytes derived from CD4-Cre PRR cKO mice was significantly lower than that derived from control mice (**Figure 1I**), indicating that PRR controls the cellular mechanisms that promote thymocyte development in mice. Adequate TCR signaling is a prerequisite for survival during positive selection. A reduction in phosphorylated LCK levels was observed in the SP cells of CD4-Cre PRR cKO mice (**Figure 1J**). Moreover, in line with the reduced thymocyte population, the expression of the anti-apoptotic protein Bcl-2 was diminished in CD4-Cre PRR cKO mice (**Figure 1K**). These results indicate that PRR promotes T cell development by enhancing TCR signaling and cell survival.

### 3.2 PRR promotes T cell maintenance in peripheral organs

**Figure 2:**
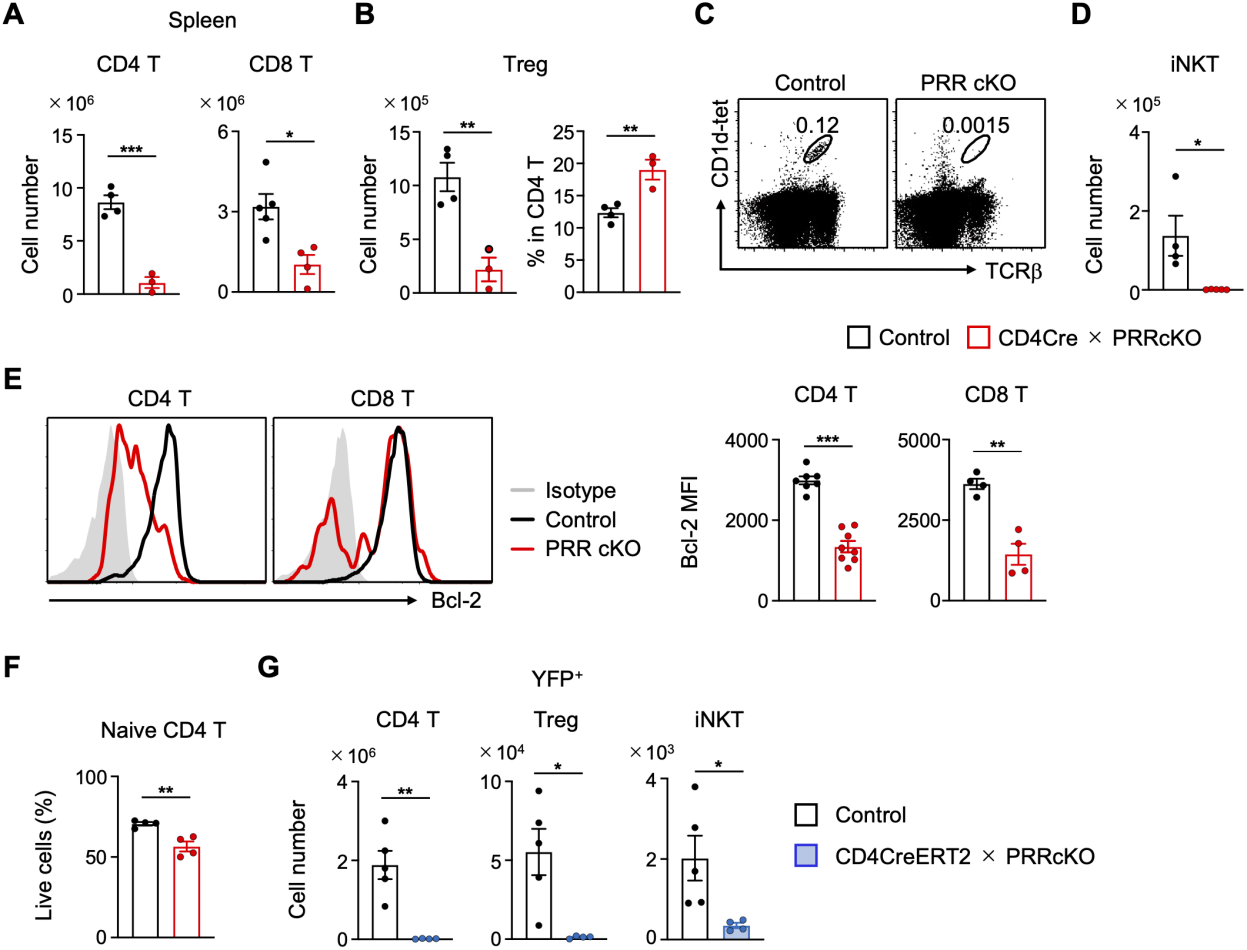
The survival of T cell subsets is impaired in the periphery of PRR-deficient mice. **(A)** Numbers of CD4 T (CD4^+^TCRβ^+^) (n = 3-4) and CD8 T (CD8^+^TCRβ^+^) (n = 4-5) cells in the spleens of control and CD4-Cre PRRcKO mice. **(B)** Number of CD25^+^CD4^+^TCRβ^+^ cells and frequency of Tregs relative to CD4 SP cells in the thymus of control and CD4-Cre PRRcKO mice (n = 3-4). **(C)** Representative FCM plots of CD1d-tetramer and TCRβ expression in splenocytes of control and CD4-Cre PRRcKO mice. **(D)** Number of iNKT (CD1d-tetramer^+^TCRβ^+^) cells in the spleens of control and CD4-Cre PRRcKO mice (n = 4-5). **(E)** Representative FCM histogram and MFI of Bcl-2 in naïve (CD44^low^) CD4 T cells (n = 7-8) and naïve CD8 T cells (n = 4) in the spleens of control and CD4-Cre PRRcKO mice. **(F)** Isolated naive CD4 T cells from control and CD4-Cre PRRcKO mice were cultured in medium without stimulation for 20 (h) Viable cell data were expressed as a percentage of the total cell number (n = 4). **(G)** Number of YFP^+^ cells in CD4 T, Treg, and iNKT cells in the spleen of CD4-CreERT2 PRRcKO mice (*CD4-CreERT2(+) Atp6ap2*^+/Y^*Rosa26*^YFP/+^) treated with tamoxifen (n = 4-5). Data are the mean ± SD with Student’s *t*-test and pooled from at least two independent experiments. ***p < 0.001; **p < 0.01; *p < 0.05; and n.s., not significant.

Next, we analyzed whether PRR promoted the survival of peripheral T cells. As observed in the thymus, the number of each T cell subset was significantly decreased in the spleens of CD4-Cre PRR cKO mice (**Figure 2A**). In addition, the number of γδ T cells increased in the spleens of CD4-Cre PRR cKO mice, as in the thymus, whereas the number of B cells remained unchanged (**Supplementary Figures S2A, B**). The number of Treg cells also decreased, but the frequency of Treg cells relative to CD4 T cells increased (**Figure 2B**), suggesting that the effect of PRR on survival varies depending on the type of T cell involved. Furthermore, the number of iNKT cells in the spleen was markedly reduced (**Figures 2C, D**). Moreover, Bcl-2 expression was diminished in splenic CD4 and CD8 T cells of CD4-Cre PRR cKO mice compared to that observed in control mice (**Figure 2E**). In accordance with the reduction in Bcl-2 expression, the viability of naive T cells in CD4-Cre PRR cKO mice declined rapidly in culture compared with that of control T cells (**Figure 2F**), indicating that PRR promotes the survival of peripheral T cells. To further confirm whether PRR regulates T cell survival after egress from the thymus, we analyzed CD4-CreERT2 PRR cKO mice. Deletion of PRR was confirmed by ROSA26-YFP, which revealed a significant reduction in YFP^+^ T cell subsets in CD4-CreERT2 PRR cKO mice following tamoxifen administration (**Figure 2G**). Additionally, the number of YFP^+^ iNKT cells was reduced in CD4-CreERT2 PRR cKO mice compared to that in control mice (**Figure 2G**), indicating that PRR supports the survival of conventional T, Treg, and iNKT cells in the periphery. Taken together, these results indicate that PRR supports the survival of peripheral T cells *in vivo*.

### 3.3 PRR promotes mitochondrial metabolism in T cells

**Figure 3:**
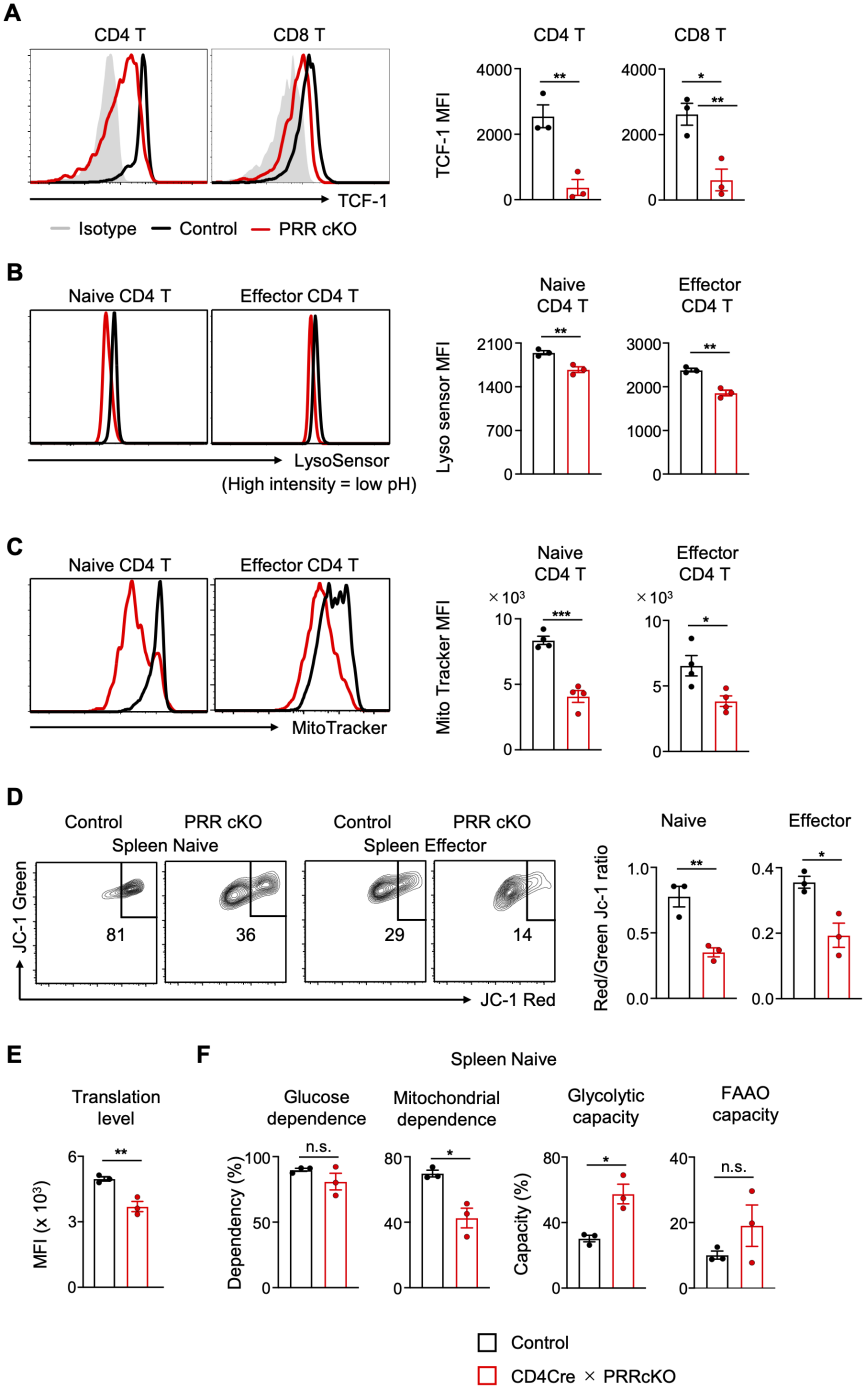
Mitochondrial metabolism is impaired in the T cells of PRR-deficient mice. **(A)** Representative FCM histogram and MFI of intracellular TCF1 staining in CD4 T and CD8 T cells in the spleen of control and CD4-Cre PRRcKO mice (n = 3). **(B)** Representative FCM histogram and MFI of LysoSensor staining of naive (CD44^low^) and effector (CD44^high^CD62L^−^) CD4 T cells in the spleens of control and CD4-Cre PRRcKO mice (n = 3). **(C)** Representative FCM histogram and MFI of MitoTracker staining of naive and effector CD4 T cells in the spleen of control and CD4-Cre PRRcKO mice (n = 3). **(D)** Representative FCM plots of JC-1 Red/Green expression and the Red/Green JC-1 ratio in naive and effector CD4 T cells in the spleen of control and CD4-Cre PRRcKO mice (n = 3). **(E)** Translation levels assessed by puromycin MFI in naive CD4 T cells of control and CD4-Cre PRRcKO mice were analyzed using the SCENITH method without 2-DG and oligomycin (n = 3). **(F)** Graphs showing the percentages of glucose dependence, mitochondrial dependence, glycolytic capacity, and fatty acid and amino acid oxidation (FAAO) capacity in naive CD4 T cells, calculated using the SCENITH method (n = 3). Data are the mean ± SD with Student’s *t*-test and pooled from at least two independent experiments. ***p < 0.001; **p < 0.01; *p < 0.05; and n.s., not significant.

PRR may promote the Wnt/TCF1 signaling pathway and lysosomal acidification. The expression of TCF1 in splenic T cells was lower in CD4-Cre PRR cKO mice (**Figure 3A**). As TCF1 upregulates its expression via the enhancer of the *Tcf7* gene locus (39), the dysfunction of TCF1 in CD4-Cre PRR cKO mice may lead to a reduction in TCF1 expression. In addition, lysosomal acidification in the T cells of CD4-Cre PRR cKO mice was impaired compared to that in control mice (**Figure 3B**). These results suggest that PRR promotes TCF1 function and maintains lysosomal pH.

Next, dysfunction of TCF1 and lysosomes in CD4-Cre PRR cKO mice may impair mitochondrial activity. In line with the finding that TCF1 increases the amount of mitochondria (40, 41), a reduction in the amount of mitochondria was observed in CD4-Cre PRR cKO mice (**Figure 3C**). Moreover, mitochondrial membrane potential is sustained by the clearance of aged mitochondria via autophagy (18). The membrane potential was lower in the T cells of CD4-Cre PRR cKO mice than in those of control mice (**Figure 3D**). This indicates that PRR promotes mitochondrial function by augmenting mitochondrial mass and membrane potential.

To determine whether PRR stimulates energy metabolism in mitochondria, we analyzed metabolic changes between control and CD4-Cre PRR cKO mouse T cells using SCENITH, a technique that elucidates metabolism-dependent translation levels by detecting the incorporation of puromycin into nascent proteins (35). Translation levels were lower in CD4-Cre PRR cKO mice, indicating impaired ATP production (**Figure 3E**). Notably, mitochondrial energy production was impaired in PRRcKO mice, whereas the dependence on glycolytic capacity increased (**Figure 3F**). Furthermore, the capacity for fatty acid and amino acid oxidation (FAAO) did not change (**Figure 3F**). These results indicate that PRR-deficient T cells depend on glycolysis for ATP production due to mitochondrial respiratory dysfunction. Collectively, these results indicate that PRR enhances mitochondrial respiration by maintaining the mitochondrial mass and membrane potential through the promotion of TCF1 activity and lysosomal acidification.

### 3.4 PRR suppresses the excessive generation of IFN-γ- and IL-17A-producing cells in the periphery

**Figure 4:**
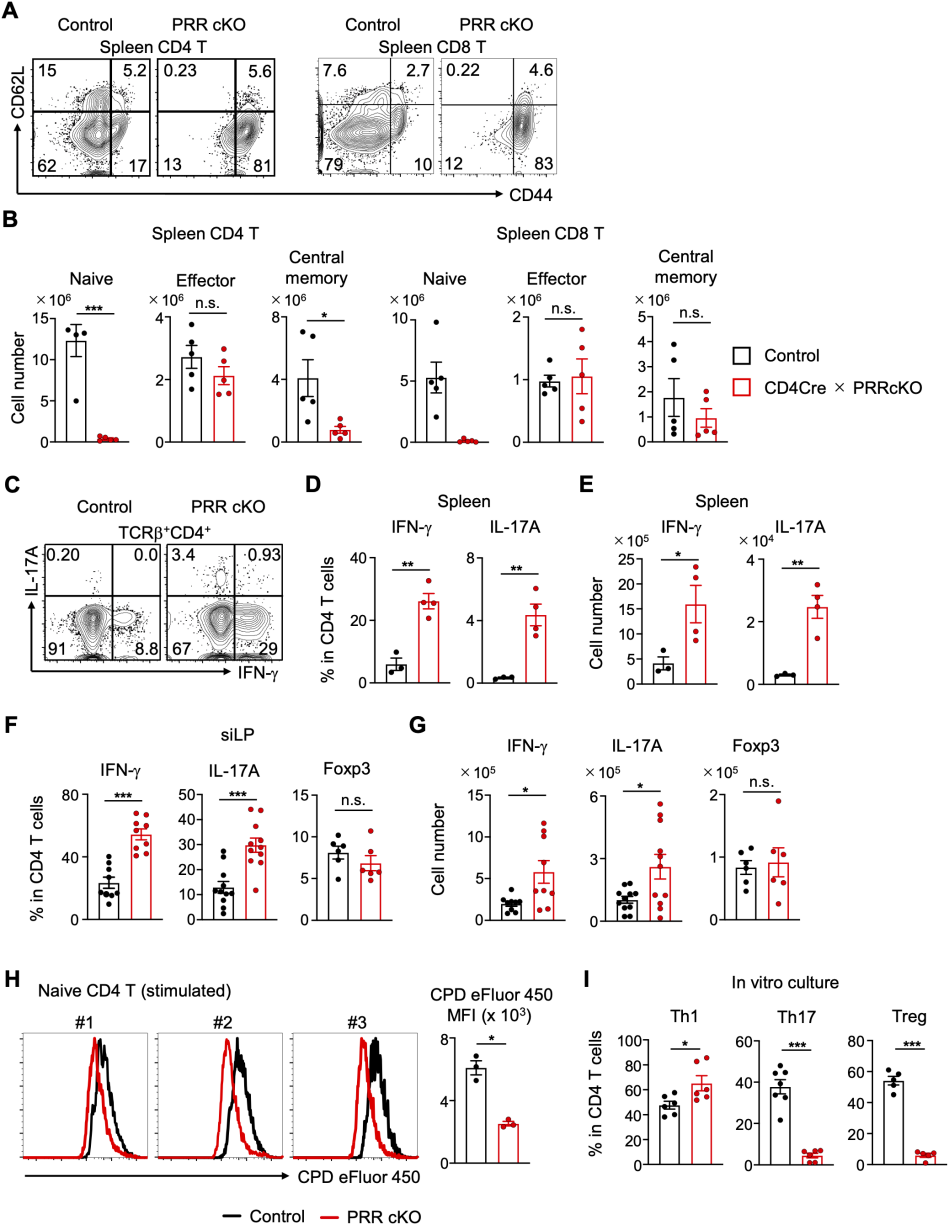
Activated and cytokine-producing T cells are increased in PRR-deficient mice. **(A)** Representative FCM plots of CD44 and CD62L expression in CD4 and CD8 T cells in the spleens of control and CD4-Cre PRRcKO mice. **(B)** Numbers of naive (CD44^low^), effector (CD44^high^CD62L^−^), and central memory (CD44^high^CD62L^+^) populations in CD4 and CD8 T cells in the spleens of control and CD4-Cre PRRcKO mice (n = 5). **(C)** Representative FCM plots of IFN-γ and IL-17A expression in splenic CD4 T cells of control and CD4-Cre PRRcKO mice. **(D)** Frequency of IFN-γ^+^ and IL-17A^+^ CD4 T cells in the spleens of control and CD4-Cre PRRcKO mice (n = 3-4). **(E)** Number of IFN-γ^+^ and IL-17A^+^ CD4 T cells in the spleens of control and CD4-Cre PRRcKO mice (n = 3-4). **(F)** Frequency of IFN-γ^+^ (n = 9), IL-17A^+^ (n = 11), and Foxp3^+^ (n = 6) CD4 T cells among the siLP CD45^+^ cells of the control and CD4-Cre PRRcKO mice. **(G)** Number of IFN-γ^+^ (n = 9), IL-17A^+^ (n = 11), and Foxp3^+^ (n = 6) CD4 T cells among siLP CD45^+^ cells of control and CD4-Cre PRRcKO mice. **(H)** Isolated naive CD4 T cells from control and CD4-Cre PRRcKO mice were labeled with CPD eFluor 450 and stimulated with anti-CD3 and anti-CD28 antibodies *in vitro* for 3 days. Triplicate histograms and MFI of fluorescence dilution (n = 3). **(I)** Isolated naive CD4 T cells from control and CD4-Cre PRRcKO mice were cultured under Th1- (n = 6), Th17- (n = 6-7), and iTreg- (n = 5) polarizing conditions. Frequency of IFN-γ^+^, IL-17A^+^, and Foxp3^+^ cells in cultured CD4 T cells under each condition. Data are the mean ± SD with Student’s *t*-test and pooled from at least two independent experiments. ***p < 0.001; **p < 0.01; *p < 0.05; and n.s., not significant.

Given the importance of mitochondrial respiration in the maintenance of naive T cells, as opposed to glycolysis, which is crucial for T cell activation (13, 14), we analyzed whether PRR controls the survival of naive and effector T cells. CD4 and CD8 T cells were fractionated into naive, effector, and central memory cells based on CD44 and CD62L expression. The majority of peripheral T cells in CD4-Cre PRR cKO mice were effector cells (**Figures 4A, B**). Consequently, the number of naive CD4 and CD8 T cells was drastically reduced, whereas the number of effector and central memory T cells remained unaltered in the spleen (**Figure 4B**). These results suggest that PRR is critical for the maintenance of naive T cells. The increase in CD44^high^ effector T cells in CD4-Cre PRR cKO mice suggests the potential for increased cytokine production by helper T cells. We observed an increase in the frequency and number of IFN-γ-producing type 1 helper T (Th1) and IL-17A-producing helper T (Th17) cells in the spleen and small intestinal lamina propria (siLP) of CD4-Cre PRR cKO mice, whereas Foxp3^+^ Treg cells remained unchanged (**Figures 4C–G**). In addition to the changes observed in subsets of effector cells, we analyzed TCRβ chain usage in the spleen to investigate whether some T cell clones expanded preferentially. We then compared the results between the young and adult mice. Although TCRβ chain usage was similar between control and CD4-Cre PRR cKO mice at an early age, older mice exhibited a more skewed TCRβ chain repertoire (**Supplementary Figures S2C, D**). This suggests that a skewed TCRβ chain repertoire may be correlated with an increase in CD44^high^ effector T cells. However, further analysis is needed to determine which TCRβ chain clones are associated with effector and inflammatory T cells.

To ascertain whether effector T cells were augmented in CD4-Cre PRR cKO mice by promoting cell proliferation, naive CD4 T cells were stimulated with anti-CD3 and anti-CD28 antibodies *in vitro*. PRR-deficient T cells exhibited enhanced proliferation *in vitro* (**Figure 4H**), indicating that PRR deficiency facilitates T cell proliferation during activation. In addition, we analyzed whether PRR facilitated the differentiation of helper T cell subsets. Consistent with the *in vivo* results, PRR-deficient T cells exhibited enhanced differentiation into IFN-γ-producing cells but failed to differentiate into Tregs (**Figure 4I**). However, the differentiation of IL-17A-producing CD4-Cre PRR cKO T cells was impaired (**Figure 4I**), indicating that the increase in Th17 cells *in vivo* may depend on homeostatic proliferation induced by cytokine surpluses (**Figures 4D–G**), resulting from a significant reduction in the number of peripheral T cells. Therefore, PRR deficiency may result in an excessive increase in inflammatory T cell subsets, such as Th1 and Th17 cells, but not in suppressive subset Treg cells, through increased proliferation.

### 3.5 PRR suppresses colitis by iNKT cell activity

**Figure 5:**
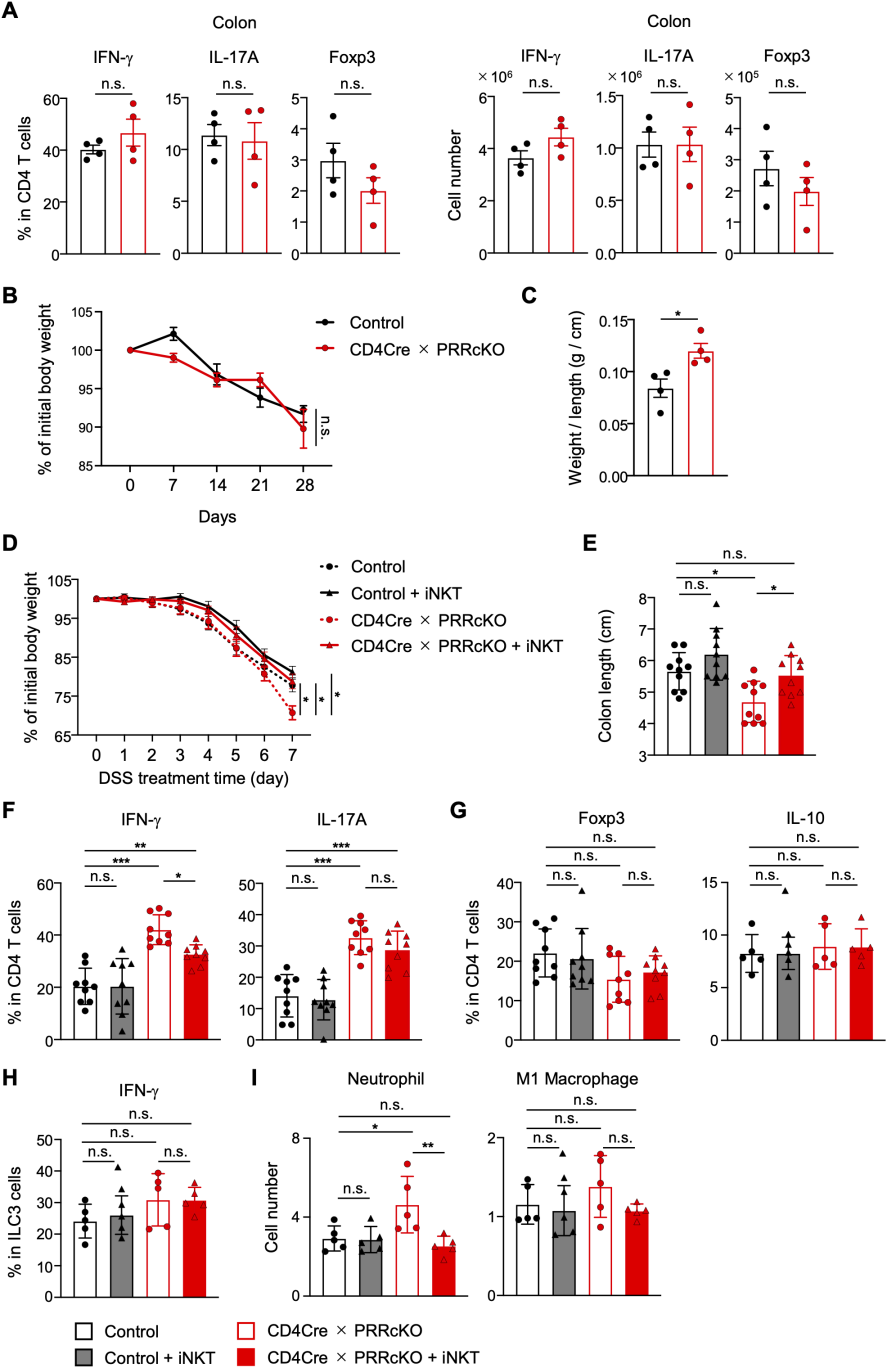
Colitis is exacerbated in PRR-deficient mice via a reduction in iNKT cells. **(A–C)** Sorted naive CD4 T cells from control and CD4-Cre PRRcKO mice were transferred into RAG2-deficient mice (n = 4). Frequency and number of IFN-γ^+^, IL-17A^+^, and Foxp3^+^ populations in CD4 T cells, which were derived from control and CD4-Cre PRRcKO mice, of colon LP CD45^+^ cells in the host mice **(A)**. Changes in the body weight of the host mice after naive T cell transfer **(B)**. Data are expressed as the percentage of the basal body weight. Weight-to-length ratio of the colon **(C)**. **(D–I)** iNKT cells were transferred or not into DSS-treated control and CD4-Cre PRRcKO mice. **(D)** Changes in the body weights of control and CD4-Cre PRRcKO mice after DSS treatment. Data are expressed as the percentage of basal body weight (n = 10). **(E)** Colon length of DSS-treated control and CD4-Cre PRRcKO mice (n = 10). **(F, G)** Frequency of IFN-γ^+^, IL-17A^+^**(F)**, Foxp3^+^, and IL-10^+^**(G)** CD4 T cells in the colon LP CD45^+^ cells of control and CD4-Cre PRRcKO mice (n = 9). **(G)** Frequency of IFN-γ^+^ ILC3 cells (CD3^−^B220^−^RORγt^+^) in the colon LP CD45^+^ cells of control and CD4-Cre PRRcKO mice (n = 5). **(I)** Number of neutrophils (Ly-6G^+^CD11b^+^) and M1 macrophages (CD11b^+^F4/80^+^CD11c^+^CD206^−^Ly6G^−^) in the colon LP CD45^+^ cells of control and CD4-Cre PRRcKO mice (n = 5). Data are the mean ± SD with Student’s *t*-test and pooled from at least two independent experiments. ***p < 0.001; **p < 0.01; *p < 0.05; and n.s., not significant.

It has been demonstrated that Th1 and Th17 cells exacerbate colitis, whereas iNKT cells mitigate colitis (27). CD4-Cre PRR cKO mice exhibited increased Th1 and Th17 cells but significantly reduced iNKT cells in the periphery (**Figures 2C, D**, **4D, E**), indicating that PRR deficiency exacerbated the colitis. We observed that approximately 9.8% of female mice spontaneously displayed symptoms such as blood in their stools or anal prolapse. First, to address whether PRR deficiency enhances the proliferation and pathogenicity of Th1 and Th17 cells in colitis, we transferred naive CD4 T cells from PRRcKO mice into RAG2-deficient mice. In the host mice, the frequency and number of IFN-γ^+^, IL-17A^+^, and Foxp3^+^ CD4 T cells in the colon remained unchanged between PRR-deficient and control T cells (**Figure 5A**). The change in body weight of host mice transferred with PRR-deficient T cells was comparable to that of the control group (**Figure 5B**), whereas the weight-to-length ratio of the colon from host mice with PRR-deficient T cells increased (**Figure 5C**). These results suggest that PRR-deficient T cells have normal proliferative ability but induce excessive inflammation, which may contribute to the promotion of colitis.

Next, to address whether iNKT cell reduction in PRRcKO mice exacerbates colitis, we treated the mice with DSS in drinking water for 7 days. To investigate whether iNKT cells mitigate colitis in CD4-Cre PRR cKO mice, we transferred iNKT cells into DSS-treated CD4-Cre PRR cKO mice. As anticipated, CD4-Cre PRR cKO mice exhibited accelerated body weight loss and colon shortening compared to control mice (**Figures 5D, E**). Furthermore, the frequencies of Th1 cells, Th17 cells, and neutrophils, but not Foxp3^+^ and IL-10^+^ CD4 T cells and IFN-γ^+^ ILC3s, were higher in CD4-Cre PRR cKO mice (**Figures 5F–I**). In contrast, the transfer of iNKT cells into CD4-Cre PRR cKO mice resulted in the alleviation of body weight loss and colon length to a similar extent as that observed in control mice (**Figures 5D, E**). Moreover, the transfer of iNKT cells into CD4-Cre PRR cKO mice did not affect the frequency of Th17 cells. However, it also reduced the frequency of Th1 cells and the number of neutrophils (**Figures 5F, I**). Since iNKT cells can suppress neutrophil infiltration into inflammatory tissues (42), these results indicate that PRR inhibits colitis by promoting the development of iNKT cells, which, in turn, suppresses IFN-γ production and neutrophil infiltration.

### 3.6 PRR enhances anti-tumor immunity by recruiting CD8 T, iNKT, and NK cells

**Figure 6:**
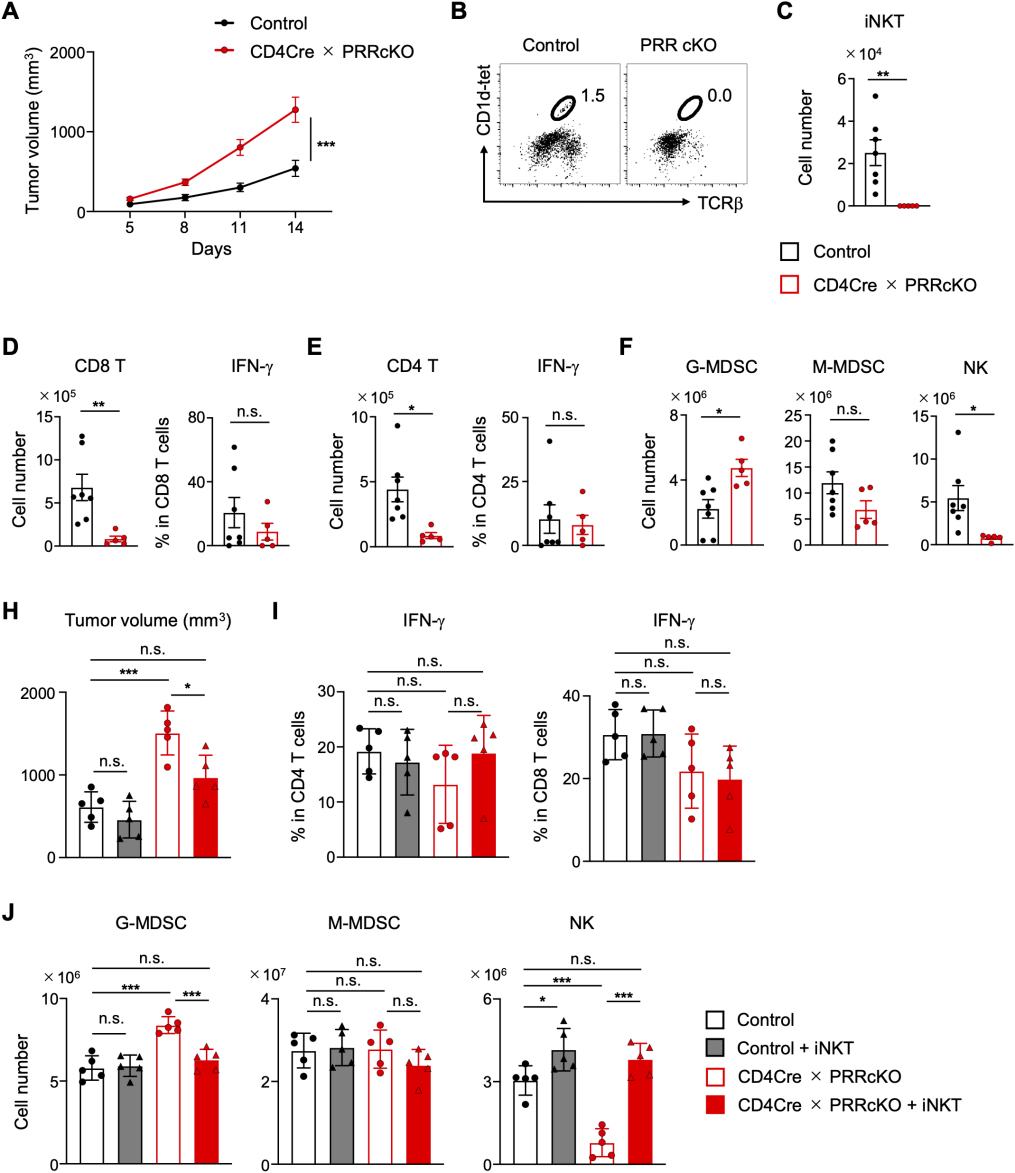
Anti-tumor immune responses are impaired in PRR-deficient mice. **(A)** Changes in B16F10 tumor volume after implantation into control and CD4-Cre PRRcKO mice (n = 5-7). **(B)** Representative FCM plots of CD1d-tetramer and TCRβ expression on tumor-infiltrating CD45^+^ cells from control and CD4-Cre PRRcKO mice. **(C)** Cell density of iNKT cells (cells/g) among tumor-infiltrating CD45^+^ cells from control and CD4-Cre PRRcKO mice (n = 5-7). **(D)** Density of CD8 T cells (cells/g) and frequency of IFN-γ^+^ populations in CD8 T cells from tumor tissue of control and CD4-Cre PRRcKO mice (n = 5-7). **(E)** Cell density of CD4 T cells (cells/g) and frequency of IFN-γ^+^ population in CD4 T cells from tumor tissue of control and CD4-Cre PRRcKO mice (n = 5-7). **(F)** Cell density of granulocyte-like MDSCs (G-MDSCs, Ly-6G^+^CD11b^+^), monocyte-like MDSCs (M-MDSCs, Ly-6G^−^CD11b^+^Ly-6C^+^F4/80^−^), and NK (NK1.1^+^CD3^−^) cells (cells/g) in CD45^+^ cells of tumor tissue from control and CD4-Cre PRRcKO mice (n = 5-7). **(H-J)** iNKT cells were transferred into B16F10-inoculated control and CD4-Cre PRRcKO mice. **(H)** B16F10 tumor volume after implantation into control and CD4-Cre PRRcKO mice (n = 5). **(I)** Frequency of IFN-γ^+^ populations in CD4 and CD8 T cells from the tumor tissue of control and CD4-Cre PRRcKO mice (n = 5). **(J)** Cell density of G-MDSCs, M-MDSCs, and NK cells (cells/g) in CD45^+^ cells of tumor tissue from control and CD4-Cre PRRcKO mice (n = 5). Data are the mean ± SD with Student’s *t*-test and pooled from at least two independent experiments. ***p < 0.001; **p < 0.01; *p < 0.05; and n.s., not significant.

iNKT cells have been demonstrated to exert anti-tumor effects through several mechanisms. These mechanisms include direct attack on tumor cells (43, 44), stimulation of CD8 T cell activation (45), and recruitment of NK cells (46). PRR deficiency enhances effector T cell development and cytokine production, which may inhibit tumor growth *in vivo*. Nevertheless, the reduction in iNKT cells due to PRR deficiency may elevate the risk of tumor development. To address this question, CD4-Cre PRR cKO mice were subcutaneously inoculated with B16-F10 melanoma cells. Tumor volumes increased in CD4-Cre PRR cKO mice (**Figure 6A**). We confirmed that iNKT cells were not observed in the tumor tissues of CD4-Cre PRR cKO mice (**Figures 6B, C**). In contrast to the observed increase in inflammatory helper T cell subsets in the periphery of the CD4-Cre PRR cKO mice (**Figures 4D–G**), the number of tumor-infiltrating CD4 and CD8 T cells was significantly lower, and the frequency of IFN-γ-producing cells was unchanged compared to that in control mice (**Figures 6D, E**). It has been reported that iNKT cells can inhibit the recruitment and proliferation of MDSCs during viral infection (47). There was an increase in granulocyte-like myeloid-derived suppressor cells (G-MDSCs) and a decrease in NK cells in the tumor tissues of CD4-Cre PRR cKO mice (**Figure 6F**).

To confirm that iNKT cell supplementation suppresses tumor growth by controlling the number of G-MDSCs and NK cells, iNKT cells were transferred into CD4-Cre PRR cKO mice that had been inoculated with B16F10 cells. As expected, tumor size decreased in CD4-Cre PRR cKO mice following the transfer of iNKT cells, whereas the frequency of the IFN-γ^+^ population in CD4 and CD8 T cells remained unchanged (**Figures 6H, I**). Furthermore, the transfer of iNKT cells reduced the number of G-MDSCs in the tumor tissue of CD4-Cre PRR cKO mice, while increasing the number of NK cells (**Figure 6J**). These results suggest that a significant reduction in iNKT cells may result in an increase in MDSCs and a decrease in NK cells in PRR-deficient mice. These results suggest that PRR enhances tumor elimination by developing iNKT cells and promoting CD4 and CD8 T cell infiltration and function against tumor growth.

## 4 Discussion

The present study indicates that PRR plays a pivotal role in the development and survival of T cells by regulating essential cellular functions, including lysosomal and mitochondrial activities. It is plausible that PRR regulates mitochondrial biogenesis and degradation by enhancing Wnt signaling or V-ATPase activity. The results demonstrated that PRR supports the survival of naive T cells, while simultaneously suppressing the proliferation of effector T cells in the periphery and inhibiting excessive cytokine production and colitis. However, elevated effector T cell levels in PRR-deficient mice were unable to effectively impede tumor growth. Moreover, PRR was indispensable for the differentiation of iNKT cells in the thymus and their survival in peripheral tissues, suppressing excessive inflammation while augmenting anti-tumor immune responses. Collectively, these results indicate that PRR plays a pivotal role in maintaining immune homeostasis, which facilitates immune responses against foreign antigens and tumor growth, while simultaneously suppressing inflammation and autoimmune diseases.

PRR deficiency in T cells significantly reduced the number of mature T cells in the thymus and peripheral organs. PRR is a multifunctional transmembrane protein that regulates V-ATPase activity and Wnt/TCF1 signaling. The severe reduction in T cells in PRR-deficient mice may result from the combined effects of PRR’s multifunctionality. V-ATPase regulates the acidification of intracellular vesicles and organelles, which is necessary for ER-to-Golgi transport, vesicle trafficking, and the recycling and degradation of proteins in lysosomes (3, 48). V-ATPase influences cell viability due to its essential role in fundamental cellular processes (3). It has been reported that the deletion of the a2-subunit isoform of V-ATPase in T cells reduces the numbers of CD4 and CD8 SP thymocytes, along with increased apoptosis of DP cells (49). Furthermore, V-ATPase increases surface Notch1 expression in DN thymocytes, facilitating Notch1-dependent proliferation of T cell progenitors (49). These findings underscore the pivotal role of V-ATPase in T cell development and survival.

PRR may enhance Wnt/β-catenin/TCF1 signaling, an important pathway for T cell development and maintenance in the thymus. Deletion of β-catenin or TCF1 in T cells reduces CD4 and CD8 SP thymocytes (50–52). β-catenin and TCF1 can upregulate the expression of anti-apoptotic factors Bcl-2 and Bcl-xL (51, 53, 54), indicating that β-catenin and TCF1 enhance T cell survival. Furthermore, β-catenin overexpression promotes the transition of DP cells to SP thymocytes (51), suggesting that β-catenin enhances positive selection in the thymus. The development of thymocytes after positive selection was impaired in the thymus of CD4-Cre PRR cKO mice, suggesting that PRR enhances the maturation of DP thymocytes into SP thymocytes via β-catenin and TCF1 signaling. LCK phosphorylation was reduced in CD4-Cre PRR cKO mice (**Figure 1J**), possibly indicating that mature thymocytes that passed positive selection decreased in CD4-Cre PRR cKO mice. Alternatively, because β-catenin can enhance TCR signaling (55), PRR may augment LCK signaling during positive selection. The reduction in mature T cells in CD4-Cre PRR cKO mice seems to be more severe than the single deletion of the V-ATPase subunit (49), β-catenin (50), or TCF1 (52), suggesting that PRR is a critical factor for T cell development in the thymus by supporting the functions of V-ATPase and Wnt/β-catenin/TCF1 signaling.

We showed that PRR also supports T cell survival in the periphery by using CD4-CreERT2-mediated deletion of PRR (**Figure 2F**). Furthermore, PRR deletion induced mitochondrial dysfunction, as evidenced by reduced amount and membrane potential. As impaired mitochondrial activity leads to cell death, mitochondrial dysfunction in CD4-Cre PRR cKO mice may contribute to the death of naive T cells. It has been reported that autophagy dysfunction due to Atg7 deletion in T cells reduces the membrane potential of mitochondria because damaged mitochondria cannot be removed (18). This impairment reduces Bcl-2 expression and induces T cell apoptosis, impairing T cell maintenance in peripheral lymphoid organs. The impairment of mitochondrial membrane potential and mitochondrial-dependent metabolism in CD4-Cre PRR cKO mice suggests that PRR promotes lysosomal acidification, which enhances autophagy, mitochondrial activity, and cell survival. Moreover, the reduction in mitochondrial mass in CD4-Cre PRR cKO mice may decrease ATP production, further impairing the survival of T cells. Considering that the Wnt/β-catenin/TCF1 pathway can increase mitochondrial amount (40, 41, 56), PRR may promote mitochondrial biogenesis via the Wnt pathway. Furthermore, it has been reported that the cytosolic release of mitochondrial phosphatase by mitophagy contributes to increased mitochondrial biogenesis (56), suggesting that PRR enhances mitochondrial biogenesis by promoting lysosomal acidification. Thus, PRR may enhance mitochondrial activity and T cell survival by promoting V-ATPase activity and β-catenin/TCF1 signaling.

In contrast to naive T cells, CD44^high^CD62L^low^ effector T cells and cytokine production by helper T cells were increased in CD4-Cre PRR cKO mice. One possibility is that homeostatic proliferation increased the relative frequency of effector T and Treg cells in the periphery of CD4-Cre PRR cKO mice due to the lymphopenic microenvironment resulting from reduced naive T cells. Furthermore, PRR-deficient T cells exhibited increased dependency on glycolysis. Since RISP ablation does not affect the homeostatic proliferation of T cells (57), this implies that the metabolic balance between OXPHOS and glycolysis controls T cell proliferation in the periphery. An alternative explanation is that mitochondrial dysfunction enhances the induction of effector T cell population. Tfam deficiency, which reduces the synthesis and stabilization of mitochondrial DNA (mtDNA), reduces peripheral T cells but increases IFN-γ- and TNF-α-producing effector CD4 T cells, exacerbating age-associated multimorbidity and DSS-induced colitis (15, 17). Furthermore, the dysfunction of lysosomes and mitophagy induces the release of mtDNA into the environment, increasing inflammatory cytokine production (17). This suggests that dysfunction of lysosomes and mitochondria in CD4-Cre PRR cKO mice may accelerate inflammatory T cell development and colitis. In contrast, anti-tumor immune responses were impaired in CD4-Cre PRR cKO mice. Mitochondrial biogenesis and the respiratory chain have been shown to enhance antigen-specific T cell responses via ROS production and augment anti-tumor immune responses by suppressing CD8 T cell exhaustion. These reports suggest that PRR suppresses inflammatory diseases but enhances tumor resistance by promoting mitochondrial and lysosomal activity.

iNKT cells were more severely reduced than other T cell subsets in CD4-Cre PRR cKO mice. Since the deletion of β-catenin or TCF1 reduces iNKT cells (58, 59), the dysfunction of β-catenin/TCF1 signaling may have contributed to impaired iNKT cell development in the thymus of CD4-Cre PRR cKO mice. Furthermore, dysfunction of mitophagy and deficiency of RISP induce a severe reduction in iNKT cells (30, 31), suggesting that the lower activity of V-ATPase in CD4-Cre PRR cKO mice reduces OXPHOS, disturbing iNKT cell development. In an experiment using CD4-CreERT2 Tg mice, we found that PRR enhanced the survival of iNKT cells in the periphery. It has been reported that iNKT cells show higher expression of genes related to the pentose phosphate pathway (PPP) and the TCA cycle but lower levels of glycolysis-related metabolites than conventional CD4 T cells (29). In addition, OXPHOS inhibition by oligomycin and FCCP strongly impairs iNKT cell survival compared to CD4 T cells (29, 30). Thus, the maintenance of iNKT cells largely depends on OXPHOS. The present study suggests that PRR enhances iNKT cell survival by promoting OXPHOS. The effects of PRR on the differentiation and survival of iNKT cells may contribute to the suppression of colitis and the promotion of anti-tumor immunity.

The present study demonstrates that PRR plays a pivotal role in supporting the development and survival of naive T and iNKT cells via the activities of TCF1 and V-ATPase, which regulate mitochondrial quality. This function of PRR may support the survival of non-pathogenic T cells but suppress the differentiation of pathogenic and inflammatory T cells. Therefore, PRR is a potential therapeutic target for preventing the development of tumors and autoimmune diseases.

## Data Availability

The raw data supporting the conclusions of this article will be made available by the authors without undue reservation.

## Supplementary material

Supplementary Figure 1 The development of T cell subset in thymus of PRR-deficient mice. **(A)** Representative thymus sections (10 μm thickness) stained with H&E in control and CD4-Cre PRRcKO mice. Scale bars, 200 μm. **(B)** Frequency of T cell populations expressing the indicated TCRβ chain in thymocytes of stage 1 (upper) and stage 2 (lower) in control and CD4-Cre PRRcKO mice (n = 4). **(C)** Representative FCM histogram of intracellular PLZF staining in iNKT (CD1d-tetramer^+^TCRβ^+^) and conventional T cells (CD1d-tetramer^−^TCRβ^+^) in the thymus of control and CD4-Cre PRRcKO mice. **(D)** Representative FCM plots of CD24 and CD69 expression in iNKT cells of control and CD4-Cre PRRcKO mice. **(E)** Number and frequency of CD24^+^CD69^+^ population in iNKT cells (n = 4). **(F)** Representative FCM plots of CD3 and γδ TCR expression in thymocytes and number of γδ T cells in control and CD4-Cre PRRcKO mice (n = 4). Data are presented as the mean ± SD and were analyzed using Student’s *t*-test and pooled from at least two independent experiments. ***, *p* < 0.001; **, *p* < 0.01; and *, *p* < 0.05.

Supplementary Figure 2 The maintenance of T cell subset in spleen of young and aged PRR-deficient mice. **(A)** Representative FCM plots of CD3 and γδ TCR expression in splenocytes and number of γδ T cells in the spleen of control and CD4-Cre PRRcKO mice (n = 4). **(B)** Representative FCM plots of CD19 and TCRβ expression in splenocytes and number of B cells in spleen of control and CD4-Cre PRRcKO mice (n = 4). (C-D) Frequency of T cell populations expressing the indicated TCRβ chain in CD4 and CD8 T cells from young (8 weeks old **(C)**) and aged (> 52 weeks old **(D)**) control and CD4-Cre PRRcKO mice (n = 4). Data are presented as mean ± SD and were analyzed using Student’s *t*-test and pooled from at least two independent experiments. *, *p* < 0.05.
