# Supplementary material for: PRR enhances anti-tumor immunity and suppresses colitis by promoting the development and survival of naive T and iNKT cells
Source: Front Immunol. 2025 Dec 10;16:1566935. doi: 10.3389/fimmu.2025.1566935 (PMC12727963; doi:10.3389/fimmu.2025.1566935)
Supplement: Supplementary Figure 1 — The development of T cell subset in thymus of PRR-deficient mice. (A) Representative thymus sections (10 μm thickness) stained with H&E in control and CD4-Cre PRRcKO mice. Scale bars, 200 μm. (B) Frequency of T cell populations expressing the indicated TCRβ chain in thymocytes of stage 1 (upper) and stage 2 (lower) in control and CD4-Cre PRRcKO mice (n = 4). (C) Representative FCM histogram of intracellular PLZF staining in iNKT (CD1d-tetramer+TCRβ+) and conventional T cells (CD1d-tetramer−TCRβ+) in the thymus of control and CD4-Cre PRRcKO mice. (D) Representative FCM plots of CD24 and CD69 expression in iNKT cells of control and CD4-Cre PRRcKO mice. (E) Number and frequency of CD24+CD69+ population in iNKT cells (n = 4). (F) Representative FCM plots of CD3 and γδ TCR expression in thymocytes and number of γδ T cells in control and CD4-Cre PRRcKO mice (n = 4). Data are presented as the mean ± SD and were analyzed using Student’s t-test and pooled from at least two independent experiments. ***, p < 0.001; **, p < 0.01; and *, p < 0.05. [file DataSheet1.pdf]

Figure S1

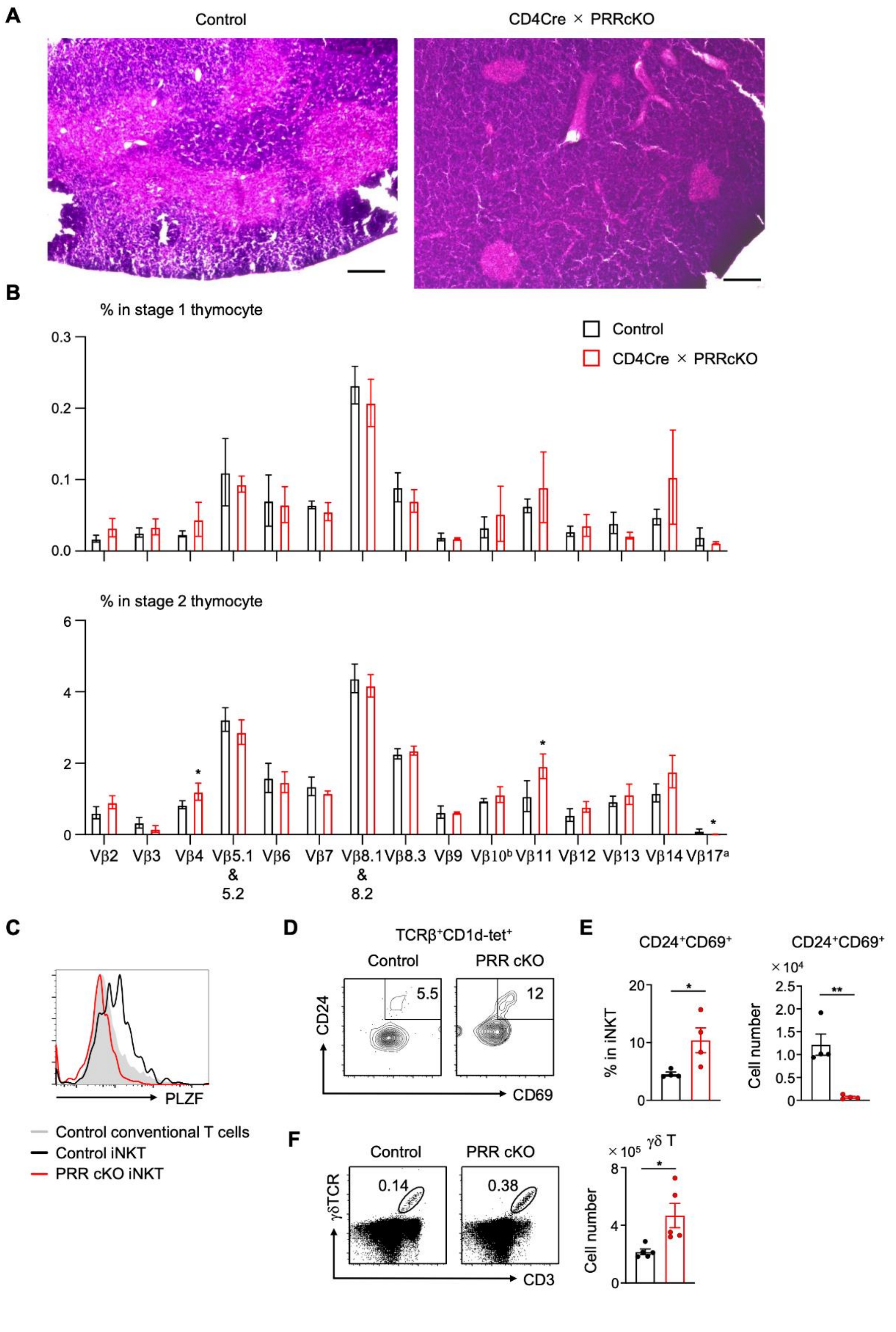
A
Control
CD4Cre × PRRcKO
B
% in stage 1 thymocyte
Control
CD4Cre × PRRcKO
% in stage 2 thymocyte
Vβ2 Vβ3 Vβ4 Vβ5.1 & 5.2 Vβ6 Vβ7 Vβ8.1 & 8.2 Vβ8.3 Vβ9 Vβ10b Vβ11 Vβ12 Vβ13 Vβ14 Vβ17a
C
PLZF
Control conventional T cells
Control iNKT
PRR cKO iNKT
D
TCRβ+CD1d-tet+
Control
PRR cKO
CD24
CD69
5.5
12
E
CD24+CD69+
% in iNKT
CD24+CD69+
Cell number
F
Control
PRR cKO
γδTCR
CD3
0.14
0.38
γδ T
Cell number

Supplementary Figure 1. The development of T cell subset in thymus of PRR-deficient mice. (**A**) Representative thymus sections (10 μm thickness) stained with H&E in control and CD4-Cre PRRcKO mice. Scale bars, 200 μm. (**B**) Frequency of T cell populations expressing the indicated TCRβ chain in thymocytes of stage 1 (upper) and stage 2 (lower) in control and CD4-Cre PRRcKO mice (n = 4). (**C**) Representative FCM histogram of intracellular PLZF staining in iNKT (CD1d-tetramer^{+}TCRβ^{+}) and conventional T cells (CD1d-tetramer^{-}TCRβ^{+}) in the thymus of control and CD4-Cre PRRcKO mice. (**D**) Representative FCM plots of CD24 and CD69 expression in iNKT cells of control and CD4-Cre PRRcKO mice. (**E**) Number and frequency of CD24^{+}CD69^{+} population in iNKT cells (n = 4). (**F**) Representative FCM plots of CD3 and γδ TCR expression in thymocytes and number of γδ T cells in control and CD4-Cre PRRcKO mice (n = 4). Data are presented as the mean ± SD and were analyzed using Student's *t*-test and pooled from at least two independent experiments. ***, $p < 0.001$; **, $p < 0.01$; and *, $p < 0.05$.

Figure S2

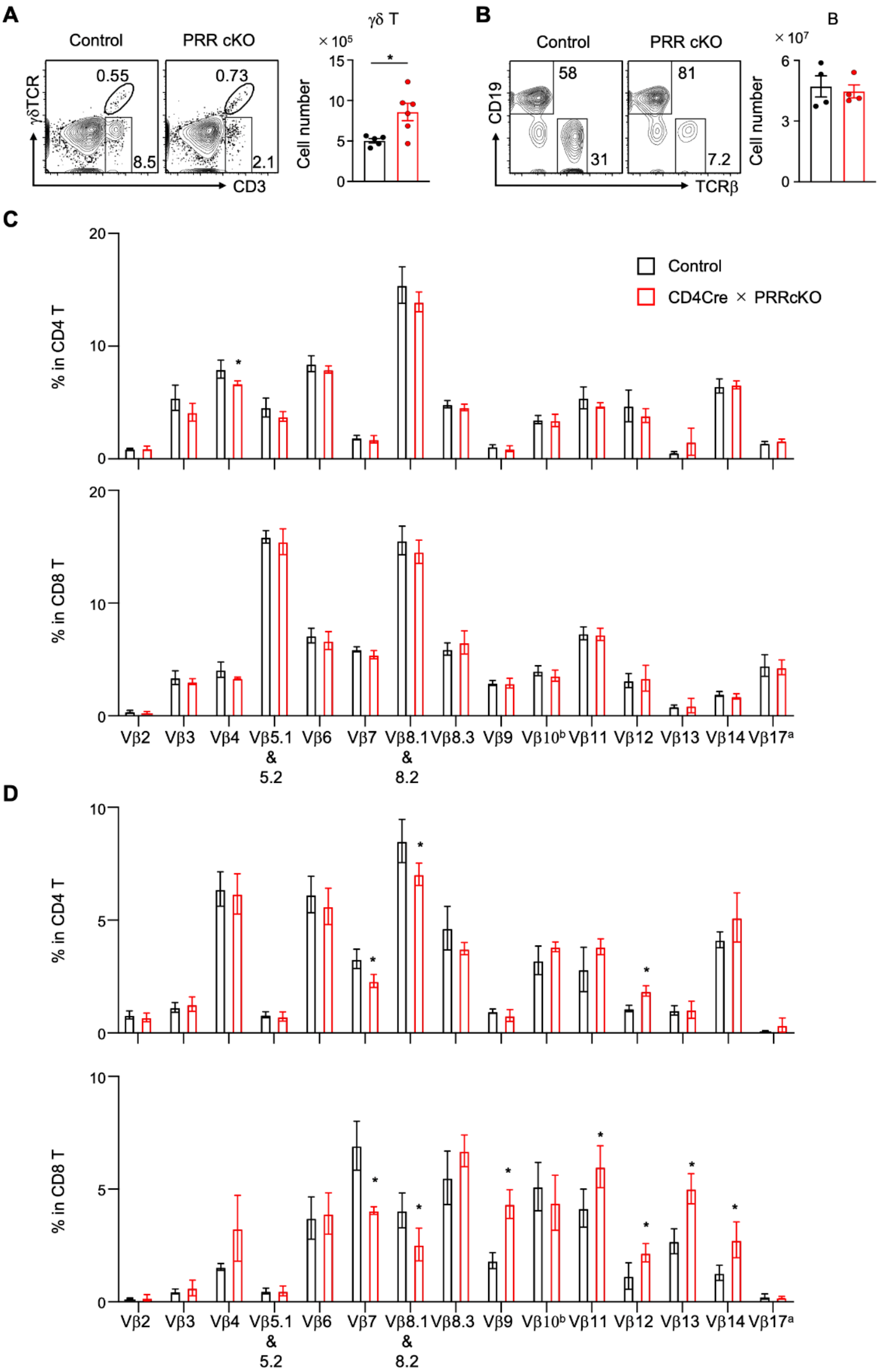
A
Control
PRR cKO
γδTCR
CD3
0.55
8.5
0.73
2.1
γδ T
× 10⁵
Cell number
15
10
5
0
*
B
Control
PRR cKO
CD19
TCRβ
58
31
81
7.2
B
× 10⁷
Cell number
6
3
0
C
Control
CD4Cre × PRRcKO
% in CD4 T
% in CD8 T
20
10
0
Vβ2
Vβ3
Vβ4
Vβ5.1 & 5.2
Vβ6
Vβ7
Vβ8.1 & 8.2
Vβ8.3
Vβ9
Vβ10ᵇ
Vβ11
Vβ12
Vβ13
Vβ14
Vβ17ᵃ
D
% in CD4 T
% in CD8 T
10
5
0

Supplementary Figure 2. The maintenance of T cell subset in spleen of young and aged PRR-deficient mice. (**A**) Representative FCM plots of CD3 and γδ TCR expression in splenocytes and number of γδ T cells in the spleen of control and CD4-Cre PRRcKO mice (n = 4). (**B**) Representative FCM plots of CD19 and TCRβ expression in splenocytes and number of B cells in spleen of control and CD4-Cre PRRcKO mice (n = 4). (**C-D**) Frequency of T cell populations expressing the indicated TCRβ chain in CD4 and CD8 T cells from young (8 weeks old (**C**)) and aged (> 52 weeks old (**D**)) control and CD4-Cre PRRcKO mice (n = 4). Data are presented as mean ± SD and were analyzed using Student's t-test and pooled from at least two independent experiments. *, $p < 0.05$.
